# Hexavalent chromium ion and methyl orange dye uptake *via* a silk protein sericin–chitosan conjugate†

**DOI:** 10.1039/c8ra03907k

**Published:** 2018-07-30

**Authors:** Swatantra Pratap Singh, Karthik Rathinam, Roni Kasher, Christopher J. Arnusch

**Affiliations:** Department of Desalination and Water Treatment, Zuckerberg Institute for Water Research, The Blaustein Institutes for Desert Research, Ben-Gurion University of the Negev, Sede Boqer Campus 84990 Israel kasher@bgu.ac.il arnusch@bgu.ac.il

## Abstract

Sericin, a protein waste product of the silk industry, was crosslinked with chitosan, and a chitosan–sericin conjugate (CS) was prepared, characterized and used to remove hexavalent chromium (Cr(vi)) ions and methyl orange (MO) dye from aqueous solutions. The CS was shown to effectively remove Cr(vi) ions and MO dye at maximum adsorption capacities (Langmuir) of 139 mg g^−1^ for Cr(vi) ions and 385 mg g^−1^ for MO dye. Moreover, the adsorption of both Cr(vi) ions and MO dye was highly pH dependent and varied under different experimental conditions. Cr(vi) ion and MO dye uptake by the CS was confirmed by attenuated total reflectance Fourier transform infrared spectroscopy, X-ray photoelectron spectroscopy (XPS) and energy dispersive spectrometry analysis. Additionally, XPS analysis of the Cr(vi)-loaded CS revealed that Cr(vi) was reduced to the less toxic Cr(iii). The CS was shown not only to be highly amenable to regeneration, but also to be able to effectively remove MO dye and Cr(vi) ions from a binary mixture.

## Abstract 2

Sericin, a protein waste product of the silk industry, was crosslinked with chitosan, and a chitosan–sericin conjugate (CS) was prepared, characterized and used to remove hexavalent chromium (Cr(vi)) ions and methyl orange dye from aqueous solutions.

## Graphical abstract

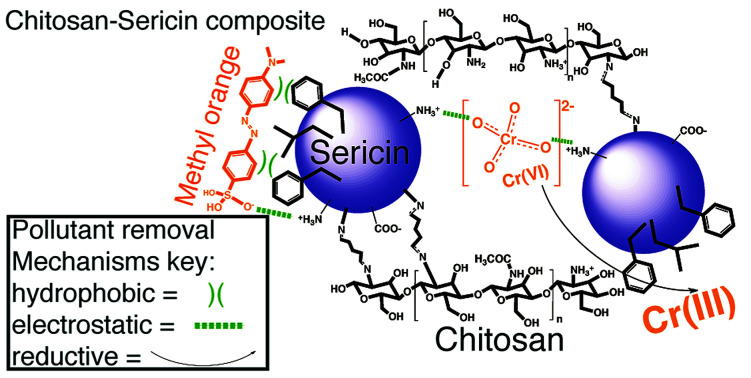

## 1. Introduction

Wastewater from the mining, chrome plating, textile, paint and pigment industries pollutes natural water sources with heavy metals and dyes, two groups of hazardous pollutants that have potentially antagonistic effects on humans and other species.^1–3^ Remediation of these hazardous pollutants can be done by using a variety of techniques, such as adsorption, precipitation, membrane processes, flocculation, ion-exchange, chemical coagulation and photo degradation.^4–13^ Among them, adsorption-based technologies are widely recognized as constituting an effective and inexpensive treatment strategy for water tainted with hazardous pollutants.^1,14,15^ In recent years, biocomposite materials^16–18^ have been used for adsorption because of their non-toxic and biodegradable nature.

For the removal of heavy metals and dyes from contaminated water, chitosan, a naturally abundant, non-toxic, biodegradable biopolymer was shown to be an effective adsorbent.^16,17,19^ Moreover, its incorporation in biocomposites, such as chitosan–polyaniline, which showed a better adsorption capacity for Cr(vi) ions than that exhibited by chitosan alone, holds even greater promise.^20,21^ Likewise, a number of other chitosan-based composites – *e.g.*, graphene oxide–chitosan,^22^ zirconium crosslinked chitosan,^23^ chitosan/silica gel,^24^ chitosan/bentonite,^25,26^ magnetic chitosan,^27^ and magnetic graphene oxide–chitosan,^28^ among others – were also shown to be effective in the adsorption of dyes and heavy metals from water. Such chitosan composites are advantageous and necessary if an insoluble adsorbent is required, since the solubility of un-crosslinked chitosan in acidic media is high.^29^

Sericin, a water soluble protein derived from the silkworm *Bombyx mori* that constitutes 25–30% of silk protein,^30,31^ is a waste product of silk processing that has been shown to be important in a variety of applications, *e.g.*, medical biomaterials and biomedicine,^32,33^ Briefly, sericin comprises 17 different types of amino acids, and it has a molecular mass >200 kD.^31^ Its main amino acids are serine, glycine and aspartic acid (∼75%), and the remainder of its composition (∼25%) comprises another 14 amino acids. In addition, sericin has both hydrophilic and hydrophobic as well as positively and negatively charged residues, which may confer on it exceptional binding capacities for heavy metals and other pollutants *via* non-covalent interactions. Similarly, Karpus *et al.* recently showed the specific binding of Cr(vi) ions by the molybdate-binding protein ModA,^34^ and Kwak *et al.* recently showed Cr(vi) removal with sericin beads.^35^ Hence, the incorporation of proteins into composites with adsorbent materials might be a viable strategy to improve the adsorption capacities of the adsorbent materials, as well as create an insoluble adsorbent from two materials that are soluble in various aqueous media. Moreover, the conversion of waste product into a valuable, environmentally friendly material has both industrial and societal benefits.

In the present study, we synthesized a chitosan and sericin conjugate *via* crosslinking with glutaraldehyde. The resulting chitosan–sericin conjugate (CS) was thoroughly characterized using spectroscopic techniques. Its adsorption performance was tested by batch experiments using hexavalent chromium (Cr(vi)) ions and methyl orange (MO) dye as models for inorganic and organic pollutants, respectively. Widely used in the leather tanning, electroplating, and metal polishing industries, the Cr(vi) ion is thought to be 1000 times more toxic than Cr(iii), and it is known to cause allergies, skin ulcerations, and damage to the livers, kidneys and blood cells. Due to its high toxicity and bioaccumulation, Cr(vi) must be removed from effluents before wastewater is discharged to the environment. To thoroughly describe the adsorption process and determine its rate, the parameters associated with the isotherm models, thermodynamic studies, and kinetic models were investigated in detail. We investigated the adsorption of Cr(vi) ions and MO dye by the CS under different experimental conditions. In brief, our findings showed significant pollutant removal and a high potential for regeneration and reuse of CS. Moreover, the influence of solution pH on the Cr(vi) ion and MO dye removal efficiencies by CS was studied. In all the pH ranges examined, CS exhibited higher selectivity for the MO dye than for the Cr(vi) ions.

## 2. Materials and methods

### 2.1. Materials

Sericin from the silkworm *Bombyx mori*, medium molecular weight chitosan, glutaraldehyde, and 1,5-diphenylcarbazide were purchased from Sigma Aldrich, Israel. Methyl orange and potassium dichromate (K_2_Cr_2_O_7_), sodium nitrate (NaNO_3_), sodium sulfate (Na_2_SO_4_), sodium bicarbonate (NaHCO_3_), sodium chloride (NaCl), sodium hydroxide (NaOH) and hydrochloric acid (HCl) were procured from Frutarom (Haifa, Israel). Deionized water (DI) was used to prepare all the solutions.

### 2.2. Preparation of CS

Solutions of chitosan (1%, w/v) and sericin (0.5%, w/v) were prepared separately by dissolving the former in acetic acid solution (2%, v/v) and the latter in DI water. Thereafter, the chitosan and sericin solutions were mixed together and stirred for 1.5 h until a homogeneous solution was obtained. Glutaraldehyde (2.5%) was then added to the solution, the solution was mixed, and the reaction mixture was kept at 0–4 °C for 24 h to ensure maximum crosslinking. After 24 h, the resultant CS conjugate was filtered and thoroughly washed with DI several times to remove unreacted sericin and glutaraldehyde. After it was dried in a hot air oven at 40 °C for 72 h, it was ready for use in further experiments.

### 2.3. CS characterization

The CS was characterized by using attenuated total reflectance-Fourier transform infra-red spectroscopy (ATR-FTIR), scanning electron microscopy (SEM), energy dispersive X-ray spectroscopy (EDX), X-ray photoelectron spectroscopy (XPS), Brunauer–Emmett–Teller (BET) surface area and thermogravimetric analysis (TGA).

The salt addition method was adopted to determine the zero point charge (pH_zpc_) of the CS in an NaCl solution (0.01 M).^36,37^ In brief, the initial pH of the NaCl solution was adjusted from pH 2 to pH 10 using 0.1 M HCl/NaOH. Thereafter, the CS (0.15 g) was added to 50 mL of NaCl solution, the mixture was equilibrated for 24 h under static conditions and its final pH was measured. The difference between the initial and final pH, *i.e.*, ΔpH, was noted and plotted against the initial pH. The pH at which ΔpH = 0 is known as the pH_ZPC_ of the CS.

### 2.4. Removal of Cr(vi) ions and MO dye using CS

Cr(vi) ions and MO dye were removed by using the batch method, and all of the adsorption experiments were done in 20 mL closed glass vials. In a typical adsorption experiment, a known amount of CS was added to 10 mL of adsorbate that comprised either MO dye at concentrations of 50–150 mg L^−1^ or Cr(vi) ions at concentrations of 50–100 mg L^−1^ solution. The resultant mixture was then agitated at 150 rpm for 60 min, after which the solution was filtered and analyzed for residual MO dye using a UV-Vis spectrophotometer at 464 nm and for residual Cr(vi) ions using the 1,5-diphenylcarbazide method.^20,38^ With the exception of the pH experiment, all the adsorption experiments were carried out at pH 7 by adjusting the initial solution pH of the adsorbate by adding either 0.1 M HCl or NaOH to the solution. The adsorption capacities and removal efficiencies were calculated by using mass balance equations [eqn (1) and (2)]:1
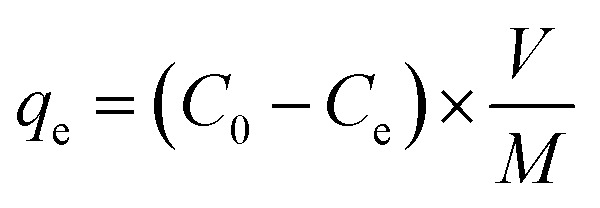

2 Removal (%) = (*C*_0_ − *C*_e_)/*C*_0_ × 100

where *q*_e_ is the equilibrium adsorption capacity (mg g^−1^), C_0_ and *C*_e_ are the initial and equilibrium adsorbate concentrations, respectively (mg L^−1^), *V* is the volume of the adsorbate solution (L), and *M* is the mass of the CS (g). Effects of pH: the effects of solution pH (3–9) on the adsorption capacities were investigated. In short, 5 mg of the CS was added to 10 mL of solution (different pH) of either 50 mg L^−1^ of MO dye or 50 mg L^−1^ of Cr(vi) ions and agitated at 150 rpm for 60 min. Effects of CS dosage: removal efficiencies of Cr(vi) ions and MO dye by the CS were investigated by using different amounts of the CS (1 to 7 mg) in a 10 mL MO dye/Cr(vi) ion solution. All experiments were done at pH 7 and under agitation for 60 min with 50 mg L^−1^ concentrations of both MO dye and Cr(vi) ions. Effects of ionic strength: The removal of MO dye (50 mg L^−1^) and Cr(vi) ions (50 mg L^−1^) by the CS was studied under different ionic strengths from 0 to 0.7 M using NaCl. The experiments were done at pH 7 with 5.0 mg of CS in 10 mL of solution with 60 min contact times. Effects of co-ions: the effects of co-ions, such as nitrate (NO_3_^−^), sulfate (SO_4_^2−^), bicarbonate (HCO_3_^−^) and chloride (Cl^−^) ions, on the removal of MO dye and Cr(vi) ions (50 mg L^−1^) by the CS were tested. NaNO_3_, Na_2_SO_4_, NaHCO_3_ and NaCl salts were used to prepare the respective stock solution of anions (1000 mg L^−1^). The adsorption experiments were carried out in the presence of these anions (50 mg L^−1^) by keeping other parameters constant as mentioned in the ionic strength experiments. Co-adsorption of MO dye and Cr(vi) ions by the CS: the co-adsorption of MO dye and Cr(vi) ions by the CS was investigated with respect to different initial pH conditions. A single solution containing 50 mg L^−1^ of both MO dye and Cr(vi) ions was prepared and the initial solution pH was adjusted from pH 3 to pH 9 by using 0.1 M HCl/NaOH. In this experiment, 5 mg of the CS was added to 10 mL of the mixed Cr(vi) ions and MO dye solution, agitated at 150 rpm for 60 min and the samples were taken for analysis. Adsorption kinetics: adsorption kinetics experiments were done at 298 K with different initial concentrations of MO dye (50, 100 and 150 mg L^−1^) and Cr(vi) ions (50, 75 and 100 mg L^−1^). Pseudo-first-order, pseudo-second-order and intraparticle kinetic models were used to fit the kinetics data. The linear forms of these kinetics models are given in the ESI in eqn (S1–S3).† Adsorption isotherms and thermodynamic studies: these studies were performed under optimized conditions at several temperatures (298, 308 and 318 K) and with varied initial concentrations of MO dye (50, 100 and 150 mg L^−1^) and Cr(vi) ions (50,75 and 100 mg L^−1^). Langmuir, Freundlich and D–R isotherm models were adapted to fit the isotherm data to explain the adsorption process. The linear forms of these isotherm models are provided in the ESI [eqn (S4–S6)].†

### 2.5. Desorption and reuse experiments

Prior to the desorption and reuse experiments, the adsorption of MO dye (50 mg L^−1^) and Cr(vi) ions (50 mg L^−1^) on CS was performed under optimized conditions. For the adsorption experiments, 0.05 g of CS was added to 100 mL of a MO/Cr(vi) ion solution and then agitated for 60 min. After the adsorption, the exhausted CS was washed with water to remove unadsorbed MO/Cr(vi) ions and then dried under vacuum. The desorption of adsorbed MO and Cr(vi) ions was done with 0.5 M NaOH for 60 min. After desorption, the CS was washed with water several times, vacuum dried and used for the next adsorption cycles.

## 3. Results and discussion

### 3.1. CS characterization

**Fig. 1:**
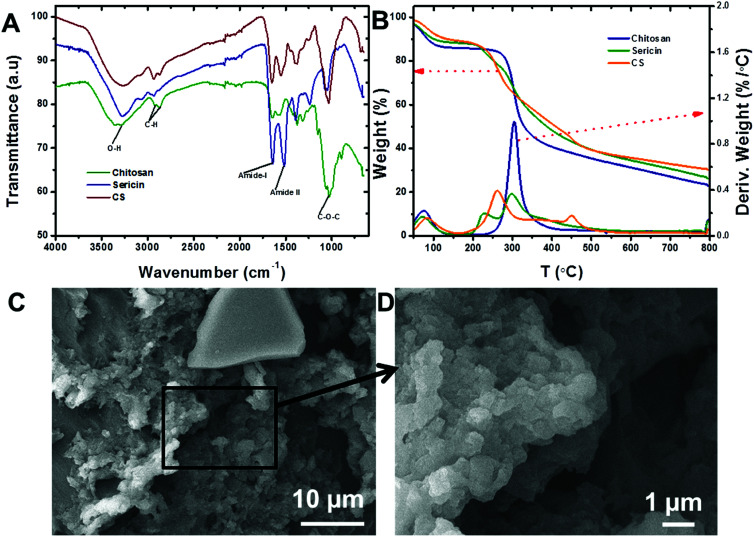
(A) ATR-FTIR spectra of chitosan, sericin and CS. (B) TGA curves for chitosan, sericin and CS. (C) Low resolution and (D) higher resolution SEM images of the CS.

Chitosan and sericin were crosslinked using glutaraldehyde, a low-cost and well-known crosslinker that reacts quickly and indiscriminately with amino groups,^39–41^ especially with amines at neutral pH, to generate thermally and chemically stable crosslinks. The resulting insoluble conjugate was washed thoroughly in order to remove any unreacted reagents. ATR-FTIR: ATR-FTIR characterization of the chitosan (Fig. 1A) showed a broad peak around 3000–3600 cm^−1^ that was attributed to the stretching vibrations of the –OH and –NH groups, while –CO–NH, –NH_2_ and –C–N groups were located at 1645, 1580 and 1315 cm^−1^, respectively. Also, the peaks that appeared at 1030 and 2920 cm^−1^ were attributed to the stretching vibrational frequencies of the –C–O and –CH_2_ groups, respectively.^42^ Similarly, the peaks observed for sericin at 3280, 1640 and 1515 cm^−1^ corresponded to the stretching vibrational frequencies of the –NH, amide I (–CO–NH) and amide II groups, respectively.^42^ In addition, peaks attributed to –OH, –NH and –C–O also appeared at 1398, 1239 and 1071 cm^−1^, respectively. Notably, after the incorporation of sericin into chitosan, the peak corresponding to –NH_2_ in chitosan at 1580 cm^−1^ shifted to 1552 cm^−1^ and new peaks corresponding to –OH and –NH (sericin) appeared at 1241 and 1398 cm^−1^. These observations suggested that the sericin and chitosan successfully combined to form a CS conjugate. TGA: thermogravimetric analysis (TGA) and derivative TGA curves of sericin, chitosan, and CS show that from 30 to 800 °C, chitosan exhibited three stages of degradation while both sericin and CS exhibited four (Fig. 1B). In chitosan and CS, the first stage degradation was mainly due to water evaporation while the second, third and fourth stage degradations corresponded to dehydration, depolymerization, and decomposition of the polysaccharides.^43^ Furthermore, the 74% loss in mass of sericin was accounted for *vis-à-vis* the four stages of degradation during the heating process. As for CS and chitosan, in the case of sericin, the first stage degradation, which occurred over a temperature range of 30 to 160 °C, corresponded to water loss (12%). The remaining degradation stages may have been due to the decomposition of protein structures. At 70%, the loss in mass of the CS was the lowest of the three substances tested. After CS were sericin (74%) and chitosan (78%). This analysis revealed that the incorporation of sericin into the chitosan improved the thermal stability of the latter. BET surface area and SEM: BET surface area, pore diameter and pore volume of the CS conjugate were measured to be 49.06 m^2^ g^−1^, 1.489 nm, and 0.068 cc g^−1^, respectively. The porous morphology of the CS evident in the SEM images (Fig. 1C and D) could account for its high surface area.

### 3.1. Removal of Cr(vi) ions and MO dye using CS

#### Effects of contact time and initial concentration

**Fig. 2:**
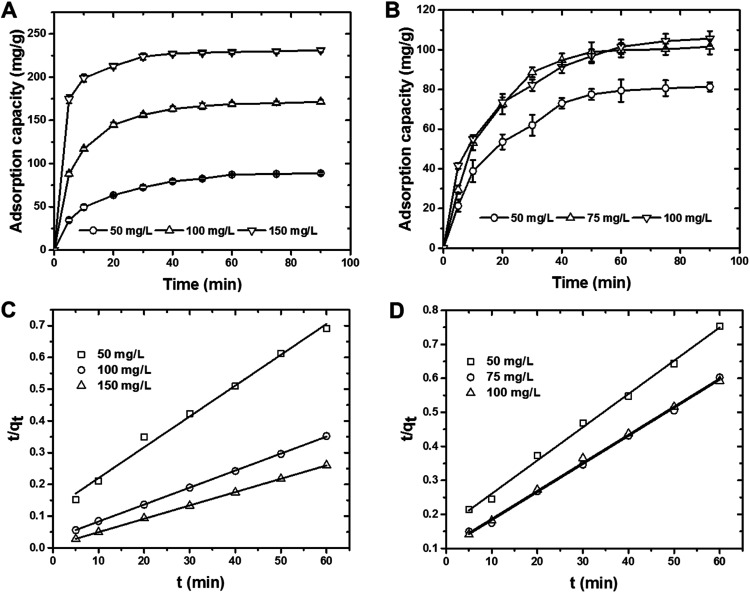
Effects of contact time and initial concentrations on the removal of (A) MO dye and (B) Cr(vi) ions by the CS (pH = 7.0, MO dye and Cr(vi) ion concentrations varied from 50–150 mg L^−1^ and 50–100 mg L^−1^, respectively, volume = 10 mL, dosage = 5 mg, and temperature = 295 K), and pseudo-second-order kinetic graph obtained for (C) MO dye and (D) Cr(vi) ions removal by the CS (pH = 7.0, MO dye and Cr(vi) ion concentrations varied from 50–150 mg L^−1^ and 50–100 mg L^−1^, respectively, volume = 10 mL, dosage = 5 mg, and temperature = 295 K).

The influence of contact time and initial concentrations of Cr(vi) ions and MO dyes on the adsorption capacities of CS were investigated and found that CS adsorption capacities were increased with the increase in initial concentration and contact time. This is probably due to the availability of higher adsorption sites the initial rate of adsorption was very fast and equilibrium was reached at 60 min for both Cr(vi) ions and MO dye (Fig. 2A and B).

#### Kinetics

The rates of the adsorption processes were depicted using pseudo-first-order, pseudo-second-order and intraparticle kinetic models. The kinetic parameters were calculated by plotting the respective linear graph of eqn (S1–S3)† (Table 1). The pseudo-second-order kinetic model exhibited the best fit with correlation coefficient (*R*^2^) values > 0.99 for Cr(vi) ions and MO dye for all cases with CS (Fig. 2C and D).

**Table 1:** Kinetic parameters obtained for MO dye and Cr(vi) ion removal using CS

Kinetic models	Parameters	MO dye			Cr(vi) ions		
		50	100	150	50	75	100
		mg L^−1^	mg L^−1^	mg L^−1^	mg L^−1^	mg L^−1^	mg L^−1^
Pseudo-first-order	*k*_ad_ (min^−1^)	0.07	0.107	0.115	0.106	0.102	0.087
	*R* ^2^	0.913	0.943	0.963	0.839	0.932	0.831
Pseudo-second-order	*q* ^cal^_e_ (mg g^−1^)	103.1	185.2	238.1	102.4	120.5	123.4
	*q* ^exp^_e_ (mg g^−1^)	87.8	170.4	230.5	79.7	99.68	101.5
	*k* × 10^−3^ (g mg^−1^ min^−1^)	0.8	0.9	2.3	0.5	0.7	0.6
	*R* ^2^	0.993	0.999	0.999	0.996	0.998	0.998
Intra-particle	*k*_i_ (mg g^−1^ min^−0.5^)	14.74	71.45	168.9	6.07	16.6	16.24
	*R* ^2^	0.981	0.900	0.861	0.969	0.935	0.967

#### Effect of pH

**Fig. 3:**
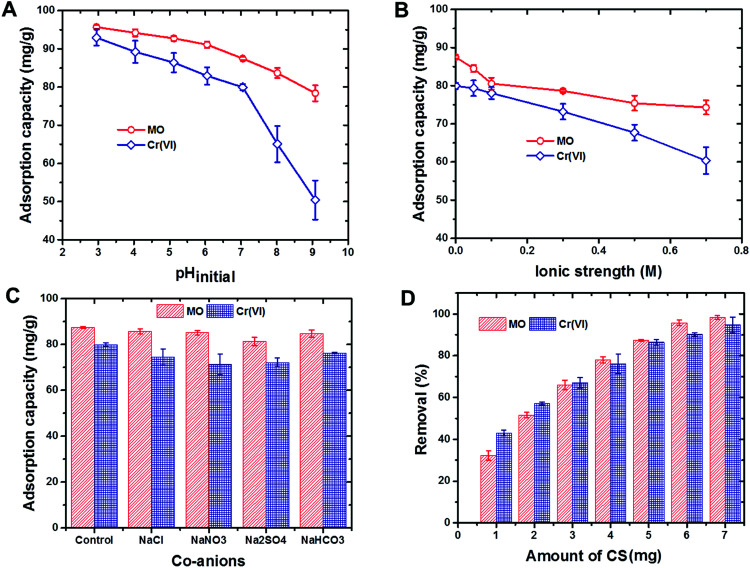
(A) Influence of solution pH on the removal of MO dye and Cr(vi) ions by the CS (MO dye and Cr(vi) ion concentrations = 50 mg L^−1^, contact time = 60 min, volume = 10 mL, dosage = 5 mg and temperature = 295 K). (B) Influence of solution ionic strength on the removal of MO dye and Cr(vi) ions by the CS (MO dye and Cr(vi) ion concentrations = 50 mg L^−1^, contact time = 60 min, volume = 10 mL, dosage = 5 mg, NaCl concentration = 0 to 0.7 M and temperature = 295 K). (C) Influence of co-existing ions on the removal of MO dye and Cr(vi) ions by the CS (pH = 7.0, MO dye and Cr(vi) ion concentrations = 50 mg L^−1^, contact time = 60 min, volume = 10 mL, dosage = 5 mg and temperature = 295 K). (D) Effect of CS dosage on the removal of MO dye and Cr(vi) ions (pH = 7.0, MO dye and Cr(vi) ion concentrations = 50 mg L^−1^, contact time = 60 min, volume = 10 mL, dosage = 1 to 7 mg and temperature = 295 K).

An important parameter that can significantly affect the adsorption process, pH is shown in Fig. 3A for Cr(vi) ions and MO dye by the CS. The removal of Cr(vi) ions and MO dye by CS decreased with the increase in solution pH (Fig. 3A). The adsorption capacity for MO dye and Cr(vi) ions changed from 95.6 and 92.9 mg g^−1^ to 78.4 and 50.4 mg g^−1^, respectively, with the increase in pH from 3 to 9. In addition, the pH_ZPC_ of the CS was determined as 5.06 using the salt addition method (Fig. S1†). The CS surface charge, which became more negative at pH values higher than 5.06, thus led to stronger electrostatic repulsions between the CS surface and the Cr(vi) ions (HCrO_4_^−^) or the anionic MO dye. This finding might be the result of the decreased adsorption capacity of CS in addition to its competition with the OH^−^ ions for the same adsorption sites. Similarly, at pH values lower than 5.06, the CS surface charge became more positive and led to stronger electrostatic interactions between the CS surface and the Cr(vi) ions/MO dye. After adsorption, pH of the treated solution was measured and found to be in the range of 5.0–6.0.

#### Effects of co-ion and ionic strength

Because the variable ionic strength of industrial effluents may interfere with the adsorption, the effects of co-ions and of ionic strength were also evaluated in the presence of several ions. The adsorption capacities of CS for Cr(vi) ions and for MO dye decreased with the increase in ionic strength (Fig. 3B). The weakening in the electrostatic attraction between the adsorbate and adsorbent may occur due to the decrease in surface and zeta potentials as the ionic strength increased, which typically reduce the adsorption capacity of the CS.^44,45^ The adsorption capacities of the CS for Cr(vi) ions and for MO were examined in the presence of competing anions, *i.e.*, NO_3_^−^, SO_4_^2−^, HCO_3_^−^ and Cl^−^ ions, and no significant inhibition was observed in terms of adsorption capacity (Fig. 3C). This confirms that the CS maintains its selectivity toward MO dye and Cr(vi) ions even in the presence of competing anions. Fig. 3D shows the influence of CS dosage on the removal of Cr(vi) ions and MO dye. Increasing the CS dosage from 1 to 7 mg, for example, improved the removal efficiencies from 43.0% and 32.3% to 94.7% and 98.3% for Cr(vi) ions and MO dye, respectively. This finding of greater adsorption at the higher CS dosage is the result of the increased number of sorption sites that can adsorb the Cr(vi) ions and MO dye.

#### Adsorption isotherm

The parameters for the Langmuir, Freundlich and D–R isotherm models were determined using eqn (S4–S6†)† (Table 2). The adsorption capacities form all three isotherms (*Q*^0^, *q*^max^ and *X*_m_) were increased with the temperature confirmed the endothermic nature of the adsorption processes. The *R*_L_ and 1/*n* values calculated less than one, confirmed the favorable conditions for the adsorption of Cr(vi) ions and MO dye to the CS. The Freundlich model exhibited the best fit (higher *R*^2^ value) for Cr(vi) ion removal by the CS. For MO dye, the Langmuir isotherm model was the best fit (higher *R*^2^ value). The Langmuir maximum sorption capacity of the CS for MO dye was calculated as 385 mg g^−1^, while for Cr(vi) ions it was 139 mg g^−1^ at 318 K. A comparison of the maximum adsorption capacities of the CS for MO dye and Cr(vi) ions (Tables 3 and 4) with those of other biopolymer based adsorbents found that for Cr(vi) ions, the CS has superior adsorption capacity than most of the other adsorbents in the list.

**Table 2:** Isotherm parameters obtained for MO dye and Cr(vi) ion removal using CS

Isotherm models	Parameters	MO dye			Cr(vi) ions		
		298 K	308 K	318 K	298 K	308 K	318 K
Langmuir	*Q* ^0^ (mg g^−1^)	344.8	370.3	384.6	108.7	125	138.8
	*b* × 10^−2^ (L mg^−1^)	5.9	7.7	10.8	3.2	2.3	2.4
	*R*_L_ × 10^−2^	15.6	8.5	5.8	7.6	5.2	3.9
	*R* ^2^	0.991	0.992	0.995	0.999	0.999	0.999
Freundlich	*k*_F_ (mg g^−1^) (L mg^−1^)^1/*n*^	33.8	41	52.4	55.8	56.5	56.7
	1/*n*	0.557	0.568	0.558	0.158	0.199	0.225
	*q* ^max^ (mg g^−1^)	551.9	707.6	860	105	121.8	137.2
	*R* ^2^	0.956	0.964	0.967	0.869	0.927	0.940
D–R	*X*_m_ (mg g^−1^)	221.3	236.2	246.3	103.4	115.2	124.6
	*k*_DR_ × 10^−5^ (mol^2^ J^−2^)	0.6	0.4	0.2	0.5	0.4	0.3
	*E* (kJ mol^−1^)	0.288	0.353	0.5	0.316	0.353	0.408
	*R* ^2^	0.973	0.968	0.963	0.937	0.952	0.981

**Table 3:** Comparison of the maximum adsorption capacity of the CS for Cr(vi) ions with the adsorption capacities of other biopolymer based adsorbents

Adsorbent materials	Maximum adsorption capacity (mg g^−1^)	Reference
Polyaniline–chitosan composite	165.6	20
Polyaniline–chitin composite	28.6	46
Polypyrrole–chitosan composite	78.6	47
Polypyrrole–chitin composite	35.2	48
Polyaniline–sodium alginate composite	73.3	5
Iron oxide coated cellulose/hydrotalcite composite	28.7	49
Iron oxide coated cellulose/hydroxyapatite composite	26.5	49
Calcium loaded alginate–bentonite composite	17.8	50
Cerium loaded alginate–bentonite composite	21.5	50
Zirconium loaded alginate–bentonite composite	23.1	50
Calcium loaded alginate–gelatin composite	19.4	51
Cerium loaded alginate–gelatin composite	24.5	51
Zirconium loaded alginate–gelatin composite	25.4	51
Hydroxyapatite–gelatin composite	19.1	52
Lanthanum loaded chitosan–silica gel composite	9.2	24
Chitosan coated fly ash composite	33.3	53
Zirconium crosslinked chitosan composite	175	23
Chitosan–sericin composite	138.8	This study

**Table 4:** Comparison of maximum adsorption capacity of the CS for MO dye with the adsorption capacities of other biopolymer based adsorbents

Adsorbent materials	Maximum adsorption capacity (mg g^−1^)	Reference
Chitosan–magnetic composite	758	54
Magnetic maghemite–chitosan nanocomposite	29.41	27
Chitosan/Al_2_O_3_/magnetite nanoparticles composite	417	55
Chitosan doped with graphene oxide	686.9	56
Magnetic chitosan–graphene oxide composite	398	28
Zr(vi) immobilized chitosan/bentonite composite	438.6	25
Chitosan/bentonite composite	224.8	26
Graphene oxide/chitosan aerogel	189.4	57
Chitosan/organic rectorite magnetic composite	5.56	58
Chitosan–sericin composite	384.6	This study

The thermodynamic parameters were calculated by using eqn (S7 and S8†)† (Table 5). The feasibility and spontaneous nature of the adsorption process was reflected in the negative Δ*G*^o^ values, which became less negative as the temperature increased. In addition, the positive Δ*H*^o^ values indicated that the adsorption processes were endothermic, and the positive Δ*S*^o^ values indicated increased randomness at the solid–liquid interface during the Cr(vi) ion and MO dye adsorption by CS.

**Table 5:** Thermodynamic parameters calculated for MO dye and Cr(vi) ion removal by the CS

Thermodynamic parameters		MO dye	Cr(vi) ions
Δ*G*^o^ (kJ mol^−1^)	298 K	−9.1	−8.56
	308 K	−8.7	−8.55
	318 K	−8.09	−8.55
Δ*H*^o^ (kJ mol^−1^)		23.6	8.44
Δ*S*^o^ (kJ mol^−1^ K^−1^)		0.05	0.001

Based on the removal efficiencies of the CS for both MO dye and Cr(vi) ions, CS capacity for regeneration and reuse did not significantly change, even after three successive cycles of reuse (Fig. S2†). This finding strongly supported the potential of using CS as a cost effective adsorbent for water purification applications.

### 3.2. Mechanism of Cr(vi) ion and MO dye removal by the CS

**Fig. 4:**
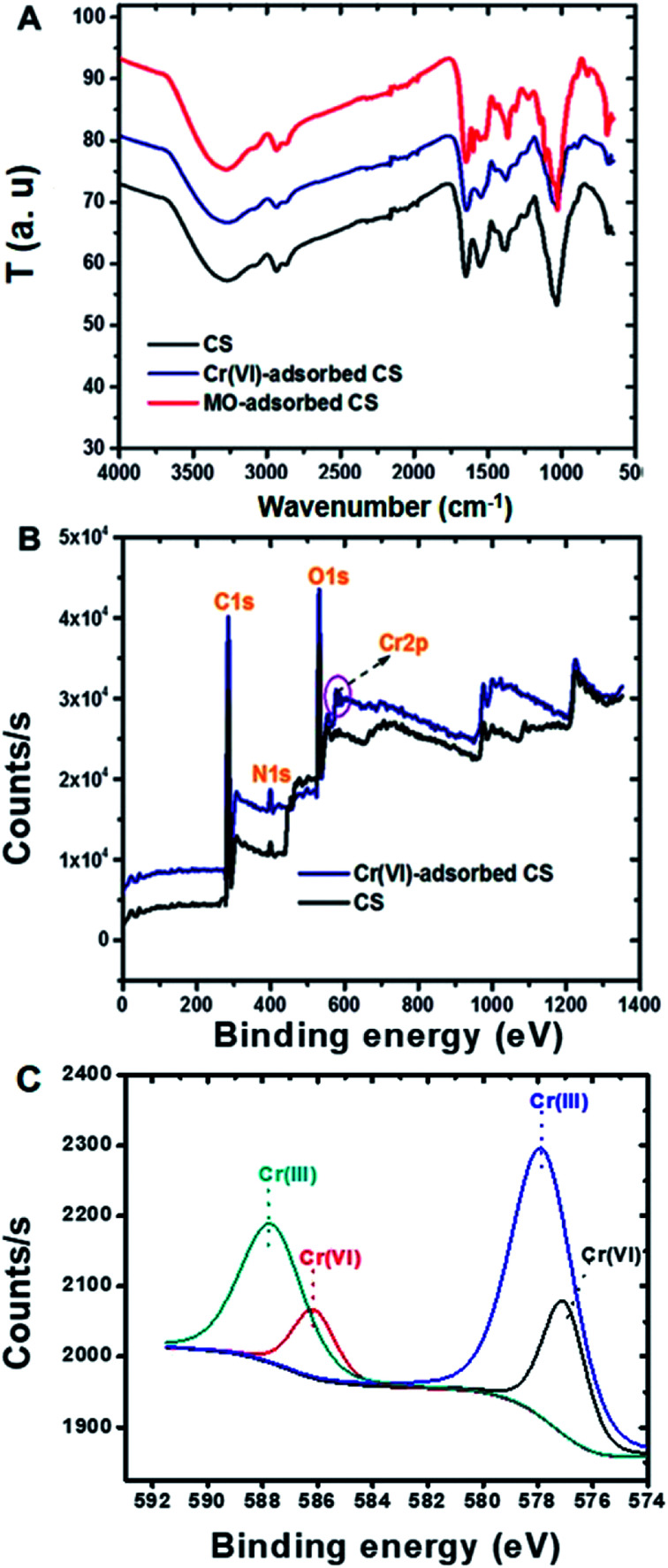
(A) ATR-FTIR spectra of the CS alone and of CS with adsorbed Cr(vi) ions and MO dye. (B) XPS general survey of the CS alone and with adsorbed Cr(vi) ions. (C) XPS spectra (de-convoluted) of CS with adsorbed Cr(vi) ions.

ATR-FTIR spectra of Cr(vi) ions and MO dye adsorbed on the CS were recorded and compared with that of the CS alone (Fig. 4A). Compared to the spectrum of CS by itself, that of CS with adsorbed MO dye showed new peaks at 824, 1113, 1366 and 1600 cm^−1^, which are assigned to C–H (bending), S=O, C–N and C=C stretching vibrations, respectively.^59^ The FTIR spectra of CS with adsorbed Cr(vi) ions show slight shifts in the –NH_2_ and –C–N peaks at 1552 cm^−1^ to 1544 cm^−1^ and 1398 cm^−1^ to 1375 cm^−1^, respectively. XPS analysis of CS with adsorbed Cr(vi) ions showed two new peaks at 587.3 and 577.9 eV that corresponded to Cr(iii) for 2p1/2 and 2p3/2, respectively (Fig. 4B and C), and this feature evidenced the reduction of highly toxic Cr(vi) in to the less toxic Cr(iii) by CS.^60^ Others have also observed the reduction of Cr(vi) with other adsorbent compositions^61–63^ and possible mechanisms have been described in detail including the direct reduction of the Cr(vi) in solution or Cr(vi) adsorption and concomitant reduction.^64^ The CS conjugate presently reported contains many electron rich groups such as hydroxyl and amine groups that might take part in the reduction process. The reduction of Cr(vi) to Cr(iii) is a great advantage with this new CS adsorbent, and this Cr(vi) adsorption on CS was further supported by EDX measurements (Fig. S3†). ATR-FTIR and XPS analyses supported that electrostatic attraction played a role in the MO dye removal mechanism by the CS, together with hydrophobic and other interactions. Whereas, the removal of Cr(vi) ions by the CS was driven mainly by electrostatic adsorption coupled with the beneficial feature of reduction. Fig. S5† is a schematic illustration depicting these possible mechanisms of Cr(vi) ion and MO dye adsorption on the CS.

### 3.3. Co-adsorption of Cr(vi) ions and MO dye by the CS from the binary mixture

The potential of the CS to remove Cr(vi) ions and MO dye from the binary mixture by co-adsorption was evaluated at different solution pH values (Fig. S4†). Cr(vi) ion and MO dye removal rates by the CS decreased with increases in solution pH, which reflects the findings of the pH study (see Section 3.1). However, in the presence of MO dye, the CS selectivity toward Cr(vi) ions was significantly influenced, while the selectivity of MO dye was not altered throughout the pH ranges studied. The competition between the Cr(vi) ions and MO dye for the same adsorption sites might have caused the observed reduction in Cr(vi) ion removal rates. These findings clearly indicate that the CS can be used for the removal of both Cr(vi) ions and MO dye from such mixtures, though the removal efficiency will be greater for the dye.

## 4. Conclusions

Sericin, a waste product of silk production, was successfully converted into a useful, environmentally friendly adsorbent with chitosan and then used for the removal of Cr(vi) ions and MO dye from aqueous solutions. P_ZPC_ and pH studies confirmed that the CS is positively charged and results in a higher adsorption capacity with decreased pH for MO dye and Cr(vi) ions. The presence of co-ions have not shown any effect on either MO dye or Cr(vi) ion removal, whereas increases in ionic strength led to decreases in adsorption capacities that were probably due to decreased surface and zeta potentials of the adsorbent. Cr(vi) ion and MO dye removal followed pseudo-second-order kinetics and the Langmuir isotherm model. The maximum CS monolayer adsorption capacities for Cr(vi) and MO dye ions were calculated as 385 and 139 mg g^−1^, respectively. These calculated values are significantly higher than those of most chitosan-based adsorbents. Electrostatic attraction was played a role in MO dye removal along with hydrophobic and other effects, whereas electrostatic adsorption coupled with reduction was the main mechanisms behind Cr(vi) ion removal. Furthermore, its reusability and its ability to simultaneously remove multiple ions make CS an excellent adsorbent for the efficient treatment of industrial effluents.

## Conflicts of interest

There are no conflicts of interest to declare.

## Supplementary Material

RA-008-C8RA03907K-s001
