# Supplementary material for: Hexavalent chromium ion and methyl orange dye uptake via a silk protein sericin–chitosan conjugate
Source: RSC Adv. 2018 Jul 30;8(48):27027–36. doi: 10.1039/c8ra03907k (PMC9083351; doi:10.1039/c8ra03907k)
Supplement: RA-008-C8RA03907K-s001 [file RA-008-C8RA03907K-s001.pdf]

Electronic Supplementary Material (ESI) for RSC Advances.
This journal is © The Royal Society of Chemistry 2018

Supporting Information

Hexavalent chromium ion and methyl orange dye uptake via a silk protein sericin-chitosan conjugate

Swatantra Pratap Singh‡, Karthik Rathinam‡, Roni Kasher*, and Christopher J. Arnusch*

Department of Desalination and Water Treatment, The Zuckerberg Institute for Water Research, The Blaustein Institutes for Desert Research, Ben-Gurion University of the Negev, Sede Boqer Campus, 84990, Israel.

*Corresponding authors e-mail: kasher@bgu.ac.il (R.K) & arnusch@bgu.ac.il (C.A)

‡Authors with equal contribution

S1. Kinetics:

S1.1 Pseudo-first-order

$$\log(q_e - q_t) = \log q_e - k_{ad}\frac{t}{2.303} \qquad \text{(S1)}$$

where q_e and q_t is the adsorption capacity (mg.g^{-1}) at equilibrium and at time t (min), respectively for the CS, k_{ad} is the equilibrium rate constant of the pseudo-first-order sorption (min^{-1}).

S1.2 Pseudo-second-order

$$\frac{t}{q_t} = \frac{1}{h} + \frac{t}{q_e} \qquad \text{(S2)}$$

where $q_t = (q_e^2 kt)/(1+q_e kt)$ is the amount of metal ions adsorbed on the surface of the CS at any time, t (mg g^{-1}), k is the pseudo-second-order rate constant (g.mg^{-1}min^{-1}), and the initial sorption rate, $h = k\, q_e^2$ (mg.g^{-1}min^{-1}).

S1.3 Intraparticle kinetic model

$$q_t = k_i t^{1/2} + C \qquad \text{(S3)}$$

where k_i (mg.g^{-1} min$^{-0.5}$) is the intraparticle diffusion rate constant and C is the thickness of the boundary layer.

S2. Isotherms:

S2.1 Langmuir isotherm

$$\frac{C_e}{q_e} = \frac{1}{Q^0 b} + \frac{C_e}{Q^0} \qquad \text{(S4)}$$

where q_e is the amount of adsorbate adsorbed per unit weight of the adsorbent (mg.g^{-1}), C_e is the equilibrium concentration of adsorbate in solution (mg.L^{-1}), Q^0 is the amount of adsorbate at complete monolayer coverage (mg.g^{-1}) and gives the Langmuir maximum sorption capacity of adsorbent, b (L.mg^{-1}) is Langmuir isotherm constant that relates to the energy of adsorption. The

essential characteristics of the Langmuir isotherm can be expressed in terms of the dimensionless constant separation factor or equilibrium parameter, $R_L = \frac{1}{1 + bC_0}$ where b ($L.mg^{-1}$) is Langmuir isotherm constant.

S2.2 Freundlich isotherm

$$logq_e = logk_F + \frac{1}{n}logC_e \quad \text{(S5)}$$

k_F is the measure of adsorption capacity ($mg.g^{-1}$) ($L.mg^{-1}$)$^{1/n}$ and 1/n is the adsorption intensity. The Freundlich maximum adsorption capacity (q^{max}) was determined using $K_F = \frac{q^{max}}{{C_0}^{1/n}}$.

S2.3 D-R isotherm

$$lnq_e = lnX_m - k_{DR}\varepsilon^2 \quad \text{(S6)}$$

X_m is the adsorption capacity ($mg.g^{-1}$) and k_{DR} is the constant related to adsorption energy ($mol^2.kJ^{-2}$). ε is Polanyi potential and can be calculated by the equation $\varepsilon = RTln(1 + \frac{1}{C_e})$ where, T is the temperature (K) and R is the gas constant (8.314 $J.mol^{-1}K^{-1}$). The value of k is used to calculate the mean of free energy E ($kJ.mol^{-1}$) of the sorption, $E = -(2k)^{-0.5}$.

S.3 Thermodynamic parameters:

The value of thermodynamic parameters was determined using the Eqs (6) and (7);

$$\Delta G^0 =- RTlnK^0 \quad \text{(S7)}$$

$$lnK^0 = \frac{\Delta S^0}{R} - \frac{\Delta H^0}{RT} \quad \text{(S8)}$$

where, ΔG^0, ΔH^0 and ΔS^0 are the standard free energy change ($kJ.mol^{-1}$), standard enthalpy change ($kJ.mol^{-1}$) and standard entropy change ($kJ.mol^{-1}K^{-1}$), respectively. K^0 is the adsorption

distribution coefficient, T is the temperature (K), R is the universal gas constant (8.314 $J.mol^{-1}K^{-1}$).

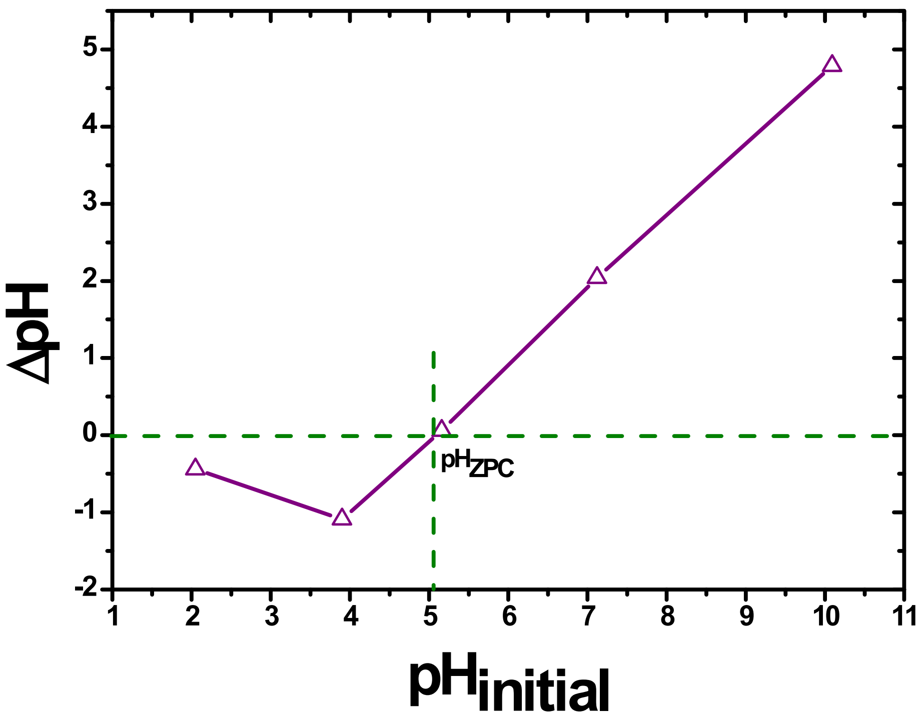

Fig. S1. pH_{ZPC} determination of the CS using salt addition method.

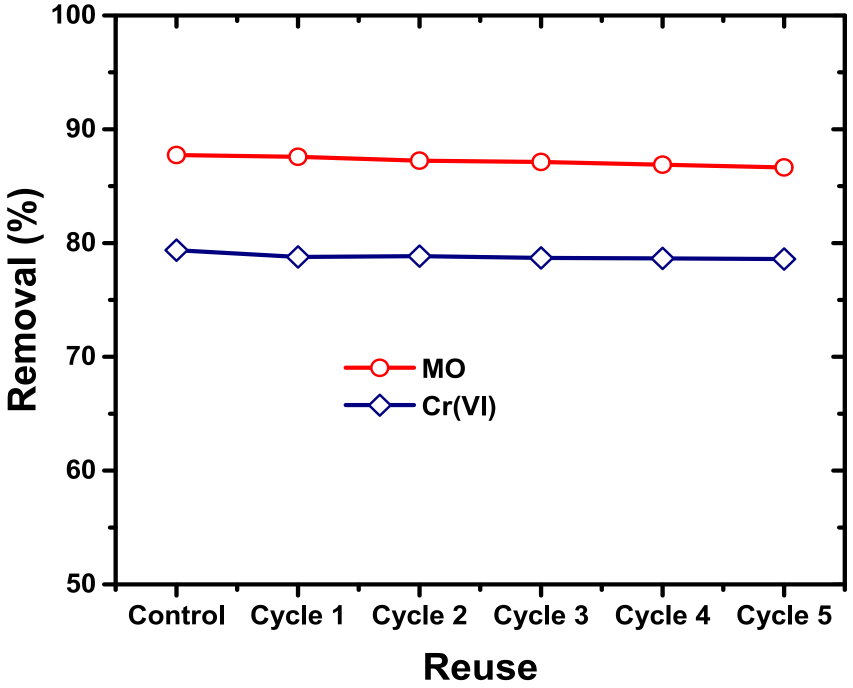

Fig. S2. Reusability analysis on MO dye and Cr(VI) ions removal using CS.

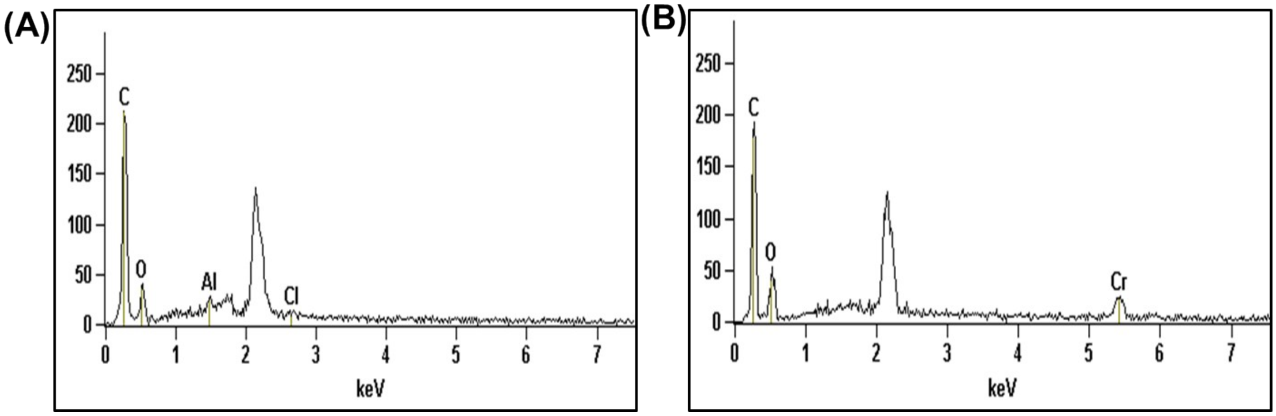

Fig.S3. EDX analysis of (A) CS and (B) Cr(VI) ions adsorbed CS.

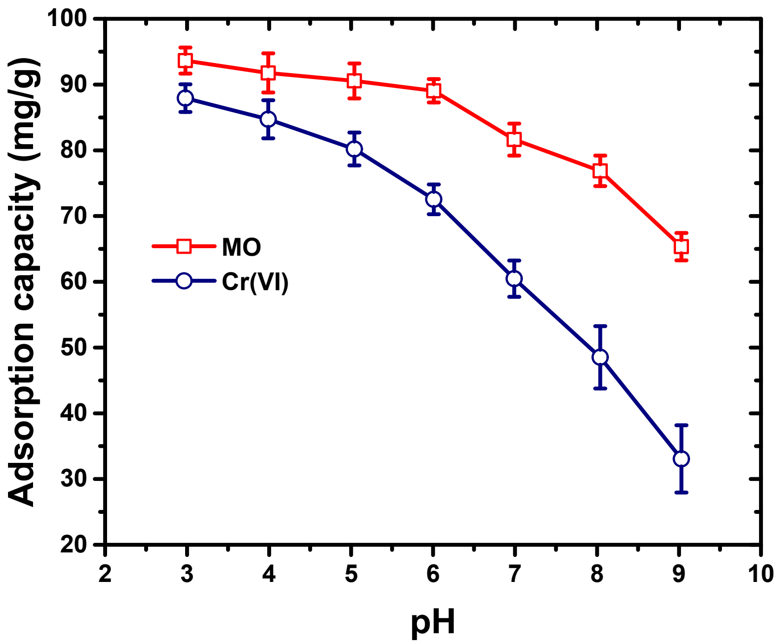

Fig.S4. Influence of pH on the coadsorption of Cr(VI) ions and MO dye using CS

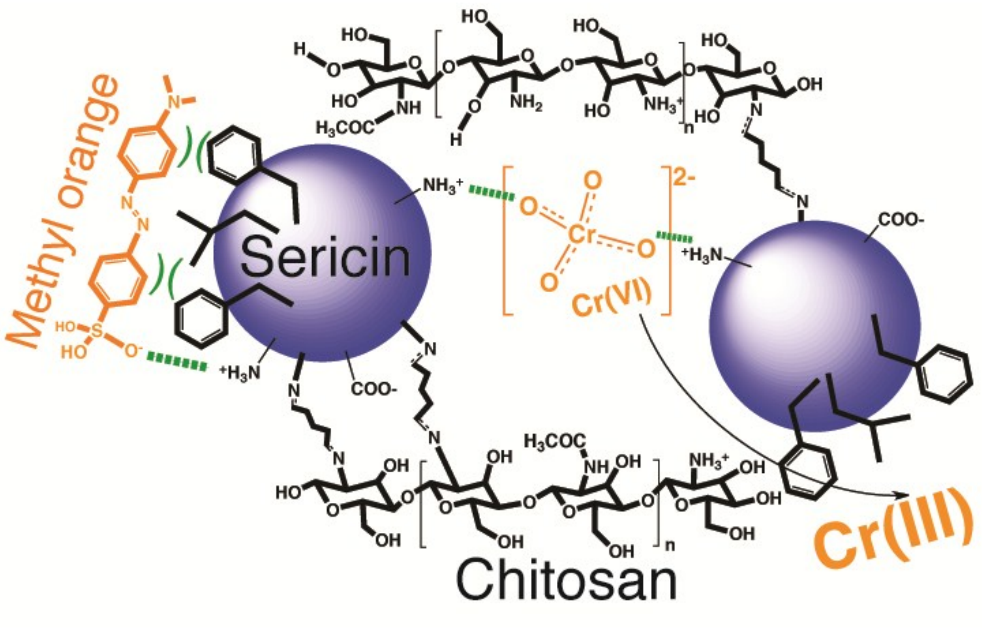

Fig.S5. Possible interactions between the CS and MO dye and Cr(VI) ions, and reduction of Cr(VI) to Cr(III). Hydrophobic interactions represented by ")(", electrostatic" represented by green dashed line.
